# Extramedullary Hematopoiesis in the Sinonasal Cavity: A Case Report and Review of the Literature

**DOI:** 10.1177/2152656720918874

**Published:** 2020-04-21

**Authors:** Carly A. Clark, Cameron P. Worden, Brian D. Thorp, Charles S. Ebert, Adam M. Zanation, Brent A. Senior, Steven M. Johnson, Wade G. McClain, Adam J. Kimple

**Affiliations:** 1 Department of Otolaryngology–Head and Neck Surgery, University of North Carolina, Chapel Hill, North Carolina; 2 Department of Pathology and Laboratory Medicine, University of North Carolina, Chapel Hill, North Carolina; 3 Marsico Lung Institute, University of North Carolina, Chapel Hill, North Carolina

**Keywords:** extramedullary hematopoiesis, sickle cell anemia, paranasal sinus, sinonasal mass, sinus tumor

## Abstract

**Background:**

Extramedullary hematopoiesis (EMH) occurs in patients with hematologic disorders, but rarely within the paranasal sinuses. We report a case of EMH in a 17-year-old male with sickle cell disease (SCD) who presented with occipital pain and sinusitis. A computed tomography (CT) scan demonstrated heterogeneous opacification of the right maxillary sinus concerning for allergic fungal sinusitis or a fungal ball with bony erosion. He was taken to the operating room for endoscopic biopsy and a limited endoscopic sinus surgery. Grossly, his maxillary sinus was filled with spiculated osseous tissue. Final pathology demonstrated active hematopoietic bone marrow filling the sinus.

**Methods:**

We present a case report and literature review of sinonasal EMH.

**Results:**

We identified 14 articles with 15 patients. EMH was typically associated with SCD or beta thalassemia. The average age of presentation was 30. There was a male sex predilection with a ratio of 11:15. The most common presenting symptom was a headache and nasal obstruction (33% for both). The most common finding on CT was a soft tissue expansile mass (73%). The most commonly affected location was the maxillary sinus (60%).

**Conclusions:**

This case report serves as a reminder to consider EMH as an uncommon cause of sinus opacification, particularly in patients with SCD or beta thalassemia. The expansion of hematopoietic tissue may be identified as a sinus mass on CT. By recognizing the potential manifestations of chronic anemia, an accurate and timely diagnosis can be made.

## Introduction

Approximately 1 in 600 African-Americans are homozygous for the sickle cell gene.^1^ This commonly inherited hematologic disorder causes sickling of red blood cells (RBCs), prompting rapid hemolysis. A common clinical manifestation of sickle cell disease (SCD) is chronic anemia. The body responds by increasing hematopoiesis. RBC production classically occurs in the bone marrow of the long bones, pelvis, spine, and sternum.^1^ With chronically elevated erythropoietin levels, organs such as the spleen and liver help augment the body’s RBC supply.^2^ These organs are areas of fetal erythropoiesis that do not typically contribute to physiologic RBC production in adults. Other, less commonly involved organs that have been documented as sites of extramedullary hematoposesis (EMH) include lymph nodes, paravertebral regions, intra-spinal canal, pre-sacral region, nasopharynx, and paranasal sinuses.^3^

There are 2 types of EMH, extraosseous and paraosseous. The extraosseous form occurs in organs with multipotent stem cells, such as the spleen and liver, that can produce hematopoietic foci remote from bone marrow. This is seen in conditions that prevent effective production of RBCs within the marrow, such as myelofibrosis. Patients typically develop splenomegaly or hepatomegaly that may manifest as early satiety, bloating, pressure, or abdominal pain. In contrast to extraosseus EMH, paraosseous EMH occurs just outside of the bone due to the herniation of hyperactive marrow.^4^ This is more common in patients with SCD and thalassemias when erythroid marrow activity is high. Paraosseous EMH may remain clinically silent until there are enough cells to form a tumor-like mass associated with symptoms.

Although it is rare to see EMH within the sinonasal cavity, based on our literature review we believe this is the 16th reported case (Table 1). The presence of EMH within the sinonasal cavity is hypothesized to occur in the paraosseous form, with the herniation of marrow out of the expanding sinus wall into the sinus cavity. We present a case and discuss the challenges of diagnosis and treatment.

**Table 1.** Patients identified from the literature review.

Year	Author	Patient Age	M/F	Laterality	Comorbidity	Presenting Symptoms	CT Findings	Diagnosis/Intervention
1984	Andreou et al.^8^	10 y/o	M	Bilateral	Beta thalassemia	Seizures, progressive aphasia	Bilateral expansile masses (ethmoid and maxillary involvement)	Unknown
1995	Fernandez et al.^9^	28 m/o	M	Bilateral	SCD (HbSS)	Sinusitis, rhinorrhea, fever, and respiratory distress	Maxillary sinus opacification and enlargement	Left maxillary sinus biopsy
2000	Joseph et al.^10^	18 y/o	M	Unilateral	Beta thalassemia, SCD	Left focal motor seizure, mild anemia	Dense lesion in sphenoid sinus	Transnasal biopsy
2001	Vargas et al.^11^	71 y/o	F	Unilateral	Myelofibrosis	Nasal mass, recurrent epistaxsis	Expansile mass in maxillary sinus	Endoscopic biopsy
2002	Kearney and Nasser^5^	24 y/o	M	Bilateral	Beta thalassemia major	Progressive weakness in lower extremities, urinary retention, and constipation	Masses in the maxillary and sphenoidal sinuses	Biopsy
2003	Rizzo et al.^12^	68 y/o	F	Bilateral	Paget’s disease	Left-sided diplopia and exophthalmos	Expansile sphenoid mass with destruction of bony septa	Biopsy
2004	Brennan et al.^13^	72 y/o	M	Unilateral	Myeloproliferative disorder	Nasal obstruction	Unremarkable	Polyp removal and biopsy
2005	Collins et al.^6^	13 y/o	M	Bilateral	SCD (HbSS)	Nasal obstruction, bifrontal headache, rhinorrhea, facial pain	Opacification of maxillary and anterior ethmoids sinus with intrasinus calcifications	Endoscopic sinus surgery and biopsy
2007	Ittipunkul et al.^14^	13 y/o	F	Bilateral	Beta thalassemia, HbE	Progressive vision loss, marked pallor	Expansile mass from ethmoid into sphenoid sinus	Monthly blood transfusion and low-dose radiotherapy (clinical diagnosis)
2008	Stamataki et al.^2^	12 y/o	M	Unilateral	SCD	Nasal obstruction	Right maxillary sinus mass obstructing the right osteomeatal complex	Right endoscopic sinus surgery and biopsy
2010	Bizzoni et al.^15^	30 y/o	M	Unilateral	Idiopathic thrombocytopenia	Frequent epistaxsis, right nasal fossa obstruction, frontal headache	Expansile mass from maxillary sinus with bony remodeling	Transnasal endoscopic biopsy and complete resection
2010	Bizzoni et al.^15^	29 y/o	M	Unilateral	Intermediate beta thalassemia	Left nasal fossa obstruction, vertex headache, fever	Expansile lesion from posterior ethmoid into sphenoid sinus	Drainage of fluid with histologic analysis
2011	Dorton and Mims^16^	41 y/o	F	Bilateral	Beta thalassemia	Recurrent epistaxsis	Diffuse marrow expansion with complete obliteration of maxillary sinus	Manage epistaxsis (clinical diagnosis)
2012	Sklar et al.^17^	14 y/o	M	Unilateral	SCD	Frontal headache	Expansile mass from sphenoid sinus	Biopsy
2014	Reiersen et al.^7^	4 y/o	M	Bilateral	SCD (HbSS)	Left-sided headache, pain, rapid progression of periorbital swelling, bilateral proptosis	Bilateral expansive maxillary heterogenous soft tissue opacification	Biopsy and exchange transfusion

Abbreviations: CT, computed tomography; HbE, hemoglobin E; HbSS, homozygous sickle cell disease; SCD, sickle cell disease.

## Case Presentation

A 17-year-old African-American male with SCD presented to his primary care physician with a 1-month history of severe occipital head pain, left facial numbness, and left eye droop. His SCD had previously caused several emergency department admissions for pain and sickle cell crisis. Magnetic resonance imaging (MRI) was ordered to rule out a possible skull infarct secondary to a sickle cell crisis. The MRI image did not show an infarction, but the patient’s left maxillary sinus was completely opacified and enlarged compared to the contralateral side. He was referred to otolaryngology for further evaluation.

**Figure 1.**
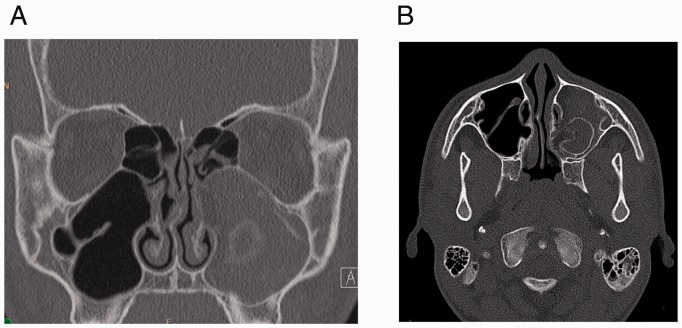
A, Coronal section showing complete opacification of the left maxillary sinus with bony expansion. Heterogeneous densities are noted centrally within the maxillary sinus. B, Axial section showing scattered foci of opacification within the left maxillary sinus.

**Figure 2.**
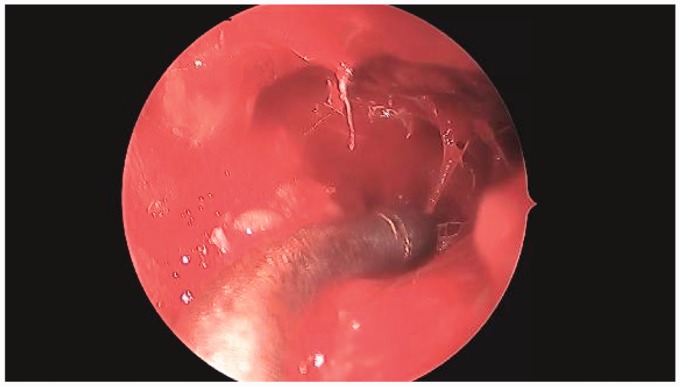
Intraoperative findings—the left maxillary sinus was filled with soft trabecular bone and fluid, without mucopurulent discharge

**Figure 3.**
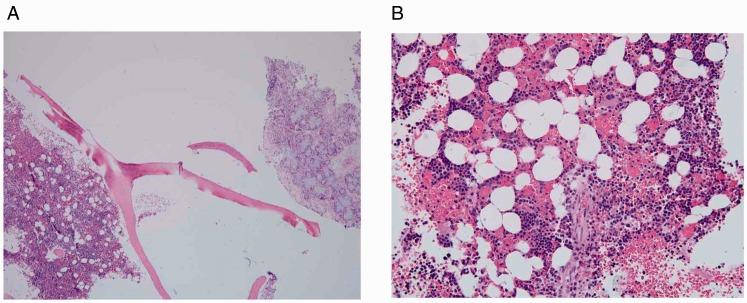
A, 40× original magnification. Inflamed sinonasal mucosa and stroma (right) intermixed with fragments of trabecular bone and normocellular marrow (left). B, 200× original magnification. Cellular bone marrow containing all 3 hematopoietic lineages (megakaryocytes, granulocytic precursors, and erythroid precursors) with erythroid hyperplasia, compatible with the patient’s active sickle cell crisis and reticulocytosis.

The patient presented to otolaryngology clinic with sinusitis and occipital pain. The physical examination was normal, but nasal endoscopy demonstrated medialization of the uncinate and medial maxillary wall within the left nasal cavity. There were no polyps or purulence noted on either side. A noncontrast computed tomography (CT) was ordered and revealed opacification and expansion of the left maxillary sinus that occluded the ostiomeatal unit consistent with a fungal ball (Figure 1(A) and (B)). Due to the persistent symptoms and unknown etiology of the sinus lesion, the patient was scheduled for endoscopic sinus surgery. Intraoperatively, the maxillary sinus was noted to filled with spiculated osseous tissue (Figure 2). A routine maxillary antrostomy was performed and specimens were collected for pathology. No drilling or special techniques were required. Pathology demonstrated erythroid hyperplasia with blood cells of all 3 hematopoietic lineages intermixed with fragments of bone (Figure 3(A) and (B)).

The patient was observed postoperatively without complications and discharged home. He has not experienced any subsequent episodes of facial pressure or pain since the surgery. Nevertheless, he continues to have frequent exchange transfusions and struggles with pain management for his underlying SCD.

## Methods

**Figure 4.**
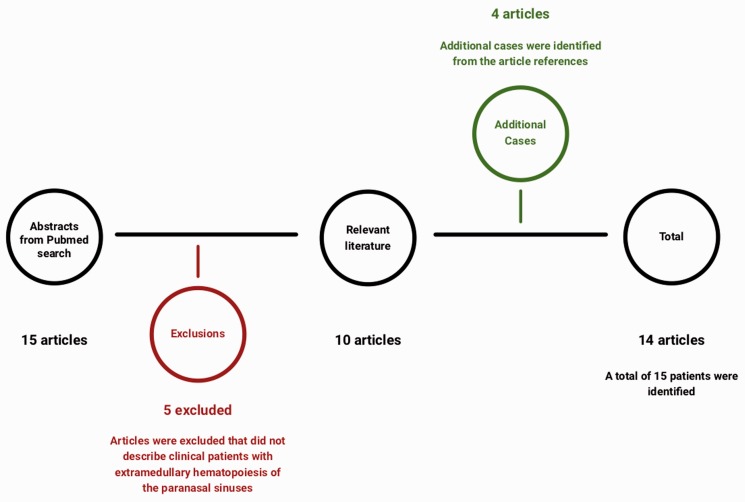
Schematic of literature review.

Data for the case report were collected from the electronic medical health record. A literature review was performed by searching the following keywords in PubMed: extramedullary hematopoiesis and paranasal sinus. Fifteen articles were identified (Figure 4). After reviewing the abstracts, 5 articles were excluded, because they did not describe clinical patients with extramedullary hematopoiesis of the paranasal sinuses. References from the remaining 10 articles were searched for additional pertinent cases and case series. We identified an additional 4 articles, for a total of 14 articles with 15 patients. A table was compiled to organize the data (Table 1).

## Results

EMH was typically associated with SCD or beta thalassemia, with an equal prevalence of each among the cases reviewed. There was a male sex predilection with 11 of 15 of the cases occurring in male patients. The average age of presentation was 30, with a minimum age of 28 months and a maximum of 72 years. The median age was 18 years. Interestingly, the 3 oldest patients had acquired conditions rather than inherited defects. Two of the patients had myeloproliferative disorders and the other had Paget’s disease.

Any sinus can theoretically be affected, but the most commonly affected location was the maxillary sinus. There were 9 cases with maxillary involvement, 6 with sphenoid involvement, and 4 with ethmoid involvement. Nasal obstruction and/or headache was a presenting symptom in 33% of patients. Recurrent epistaxis was the presenting symptom in 20% of patients. One reported case of sinonasal EMH was incidentally identified on CT scan after a motor vehicle collision.^5^

## Discussion

EMH can present as a sinus opacification in patient chronic anemia from diseases such as thalassemias, SCD, and myeloproliferative disorders. After reviewing the literature, common themes were identified in the detection and diagnosis of EMH (Table 1).^2,5–17^ On CT imaging, the hematopoietic tissue typically appears as a soft tissue mass that may demonstrate calcifications. Several cases involving the paranasal sinuses showed bone remodeling, bulging, and protrusion into other sinuses. These findings may resemble the appearance of allergic fungal sinusitis on imaging, and thus many of the pre-operative diagnoses included fungal etiologies. However, in the few cases that utilized MRIs, sinonasal EMH demonstrated signal intensity and enhancement similar to that of red bone marrow.^1^

No definitive therapeutic guidelines for paranasal sinus EMH exist. Twelve of 15 cases proceeded with a biopsy to confirm the diagnosis and to exclude neoplasm. Several authors have suggested that the hematopoietic tissue should not be removed, as it serves as a vital contributor to the patient’s RBC reserve.^6^ In addition to surgery, 2 of 15 patients were treated with exchange transfusions which serve to address the underlying anemia and decrease the demand on the extramedullary marrow.^7^ Normalization of hematocrit levels suppresses the hematopoietic foci to the extent that it no longer produces symptoms. Even in the more serious cases, with seizures, proptosis, and vision loss as presenting symptoms, patients were successfully managed with conservative management.

## Conclusions

Extramedullary hematopoiesis within paranasal sinuses is a rare diagnosis in a patient with chronic anemia. Imaging is frequently concerning for allergic fungal sinusitis. A biopsy is required to diagnose EMH and rule out an underlying malignancy. We recommend that EMH be included in the differential diagnosis of a soft tissue, expansile sinus mass presenting in patients with known hematologic conditions.
